# Supplementary material for: GSK-3α Inhibition in Drug-Resistant CML Cells Promotes Susceptibility to NK Cell-Mediated Lysis in an NKG2D- and NKp30-Dependent Manner
Source: Cancers (Basel). 2021 Apr 9;13(8):1802. doi: 10.3390/cancers13081802 (PMC8070516; doi:10.3390/cancers13081802)
Supplement: Supplementary file 1 [file cancers-13-01802-s001.zip › cancers-1124798-supplementary_Proof.docx]

**Supplementary Materials**





**Fig. S1. Inhibition of GSK-3 by LiCl enhances NK cell-mediated cytotoxicity.**

(**A** and **B**) NK cell cytotoxicity against KCL-22M cells pretreated with the GSK-3 inhibitor LiCl (10 mM; pan-GSK-3) (**A**) or TDZD-8 (1 μM; GSK-3β-selective) (**B**) for 48 h was assessed using a europium-based cytotoxicity assay with purified primary NK cells stimulated with IL-2 at the indicated effector to target (E:T) cell ratios. * *p* < 0.05; ** *p* < 0.01; *** *p* < 0.001.





**Fig. S2. Inhibition of GSK-3 by LiCl enhances the gene transcription of MICA and ULBP2.**

KCL-22M cells were incubated with LiCl (10 mM) for 24 h. The relative mRNA levels of NKG2DL were determined by quantitative real-time PCR and normalized to the levels of β-actin mRNA. Data are representative of two independent experiments and are presented as the fold change relative to untreated cells.


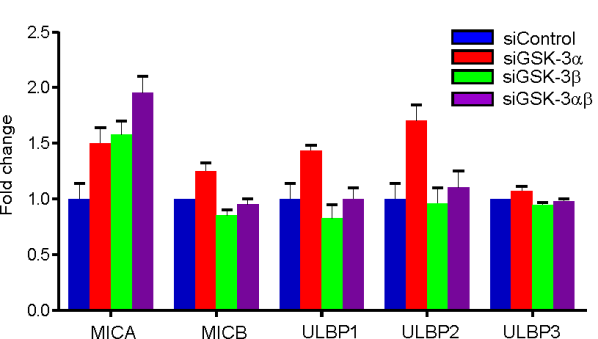


**Fig. S3. GSK-3α knockdown enhances the gene transcription of NKG2DLs.**

KCL-22M cells were transfected with control siRNA or siRNA specific for GSK-3α and/or GSK-3β for 48 h. The relative mRNA levels of NKG2DL were determined by quantitative real-time PCR and normalized to the levels of β-actin mRNA. Data are representative of three independent experiments and are presented as the fold change relative to the levels in cells transfected with control siRNA.





**Fig. S4. GSK-3 regulation of NKG2DL expression is independent of BCR-ABL mutation.**

Analysis of NKG2DL surface expression on KCL-22 cells harboring wild-type *BCR-ABL1* after siRNA-mediated knockdown of GSK-3α and/or GSK-3β for 48 h. Data are representative of three independent experiments.





**Fig. S5. GSK-3α isoform selectively regulates IFN-γ production of NK cells against KCL-22M cells.**

Intracellular IFN-γ assay with IL-2-stimulated PBMCs against KCL-22M cells depleted of GSK-3α and/or GSK-3β. Representative flow cytometry profile (*left*) and summary graph (*right*) showing the percentages of IFN-γ^+^ on CD3^-^CD56^+^ NK cells. Data are representative of three independent experiments. The mean values ± s.d. of three independent experiments are shown. *** *p* < 0.001.





**Fig. S6. Inhibition of c-Myc diminishes NKG2DL upregulation by siRNA-mediated knockdown of GSK-3α.**

Analysis of NKG2DL surface expression on KCL-22M cells after siRNA-mediated knockdown of GSK-3α in the absence or presence of the c-Myc inhibitor 10058-F4 (25 μM) for 48 h.


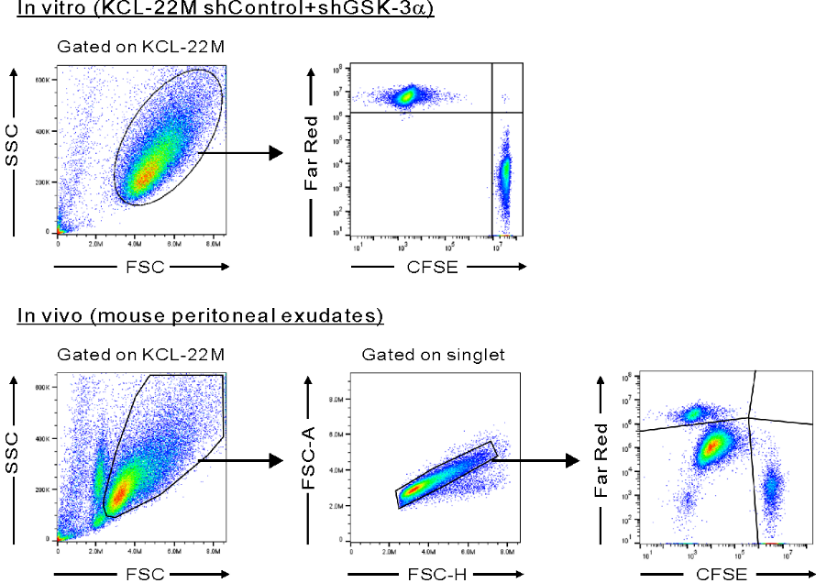


**Fig. S7. FACS gating strategy.**

Profiles showing the gating strategy for identifying the CFSE-stained KCL-22M-shControl and FarRed-stained KCL-22M-shGSK-3α cells within the gate closely matched to KCL-22M cells. Gating strategy (*in vitro*): forward scatter (FSC) vs. side scatter (SSC) (*left panel*) and CFSE vs. FarRed (*right panel*). Gating strategy (*in vivo*): forward scatter (FSC) vs. side scatter (SSC) (*left panel*), then FSC-Height vs. FSC-Area (*middle panel*), and CFSE vs. FarRed (*right panel*).


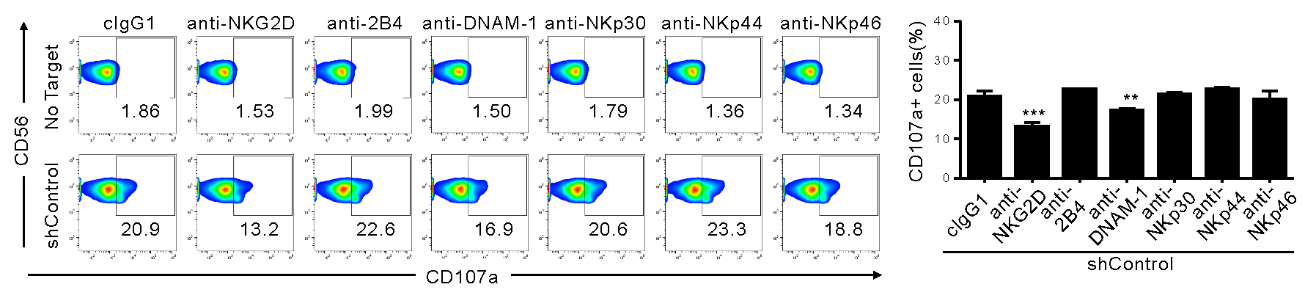


**Fig. S8. NKG2D and DNAM-1 contribute to the degranulation of NK cells against KCL-22M-shControl cells.**

KCL-22M cells transduced with control shRNA (shControl) were mixed with expanded primary NK cells preincubated with blocking Abs to the indicated receptors (20 μg/mL) for 2 h. Degranulation was measured by the expression of CD107a on CD3^-^CD56^+^ NK cells. Representative flow cytometry profile (*left*) and summary graph (*right*) demonstrating the percentage of CD107a^+^ NK cells. Values represent the means ± s.d. ** *p* < 0.01; *** *p* < 0.001.


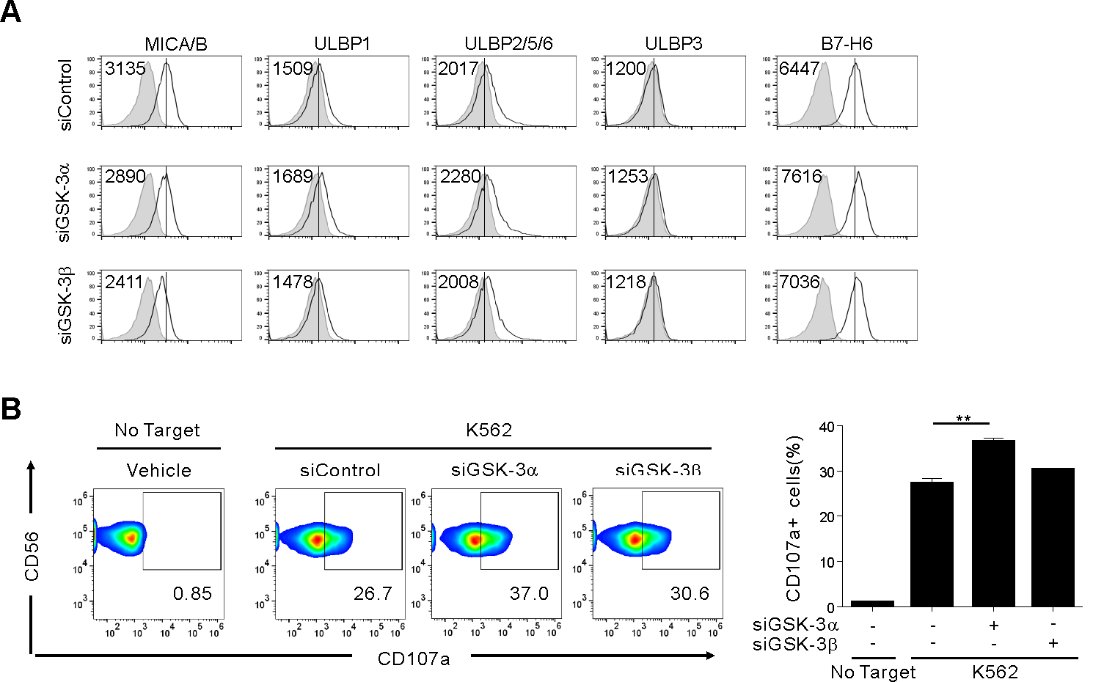


**Fig. S9. GSK-3α knockdown enhances NKG2DL and NKp30L expression on K562 cells and their cytolysis by NK cells.**

(**A**) K562 cells transfected with control siRNA or siRNAs specific for GSK-3α or GSK-3β for 48 h were analyzed for the surface expression of NKG2DL (MICA/B, ULBP1, ULBP2/5/6, and ULBP3) and NKp30L (B7-H6) by flow cytometry.

(**B**) Degranulation of NK cells against K562 cells depleted of GSK-3α or GSK-3β was measured using expanded primary NK cells. Representative flow cytometry profile (*left*) and summary graph (*right*) demonstrating the percentage of CD107a^+^ on CD3^-^CD56^+^ NK cells. Values represent the means ± s.d. ***p* < 0.01.

**Supplementary Table 1. List of antibodies used for analysis of NK cells and signaling molecules.**

| Category | Antigen | Clone | Isotype/  source | Purified/  Conjugated | Company |
| --- | --- | --- | --- | --- | --- |
| NK cell receptor, ligand, function | NKG2D | 1D11 | Mouse IgG1 | Purified | BD |
|  | MICA/B | 6D4 | Mouse IgG2a | PE | BD |
|  | ULBP1 | 170818 | Mouse IgG2a | PE | R&D Systems |
|  | ULBP2/5/6 | 165903 | Mouse IgG2a | PE | R&D Systems |
|  | ULBP3 | 166510 | Mouse IgG2a | PE | R&D Systems |
|  | CD244/2B4 | C1.7 | Mouse IgG1 | Purified | BioLegend |
|  | DNAM-1 | DX11 | Mouse IgG1 | Purified | BD |
|  | CD155/PVR | SKII.4 | Mouse IgG1 | PE | BioLegend |
|  | CD112/Nectin-2 | R2.525 | Mouse IgG1 | PE | BD |
|  | CD337/NKp30 | P30-15 | Mouse IgG1 | Purified | BioLegend |
|  | B7-H6 | 875001 | Mouse IgG1 | PE | R&D Systems |
|  | CD336/NKp44 | P44-8 | Mouse IgG1 | Purified | BioLegend |
|  | CD335/NKp46 | 9E2/NKp46 | Mouse IgG1 | Purified | BD |
|  | isotype control mouse IgG1 | MOPC-21 | Mouse IgG1 | Purified | BioLegend |
|  |  |  |  | PE | BD |
|  | isotype control mouse IgG2a | G155-178 | Mouse IgG2a | PE | BD |
|  | CD3 | SK7 | Mouse IgG1 | PerCP | BD |
|  | CD56 | NCAM16.2 | Mouse IgG2b | PE | BD |
|  | CD107a | H4A3 | Mouse IgG1 | FITC | BD |
|  | IFN-γ | 25723.11 | Mouse IgG2b | FITC | BD |
| Signaling | GSK-3α/β | D75D3 | Rabbit IgG | Purified | Cell Signaling |
|  | GSK-3β | 27C10 | Rabbit IgG | Purified | Cell Signaling |
|  | pS473-Akt | Ser473 (Polyclonal) | Rabbit | Purified | Cell Signaling |
|  | Akt | Akt (Polyclonal) | Rabbit | Purified | Cell Signaling |
|  | p-ERK1/2 | Thr202/Tyr204  (Polyclonal) | Rabbit | Purified | Cell Signaling |
|  | ERK1/2 | Erk1/2 (Polyclonal) | Rabbit | Purified | Cell Signaling |
|  | c-Myc | 9E10 | Mouse IgG1 | Purified | Santa Cruz |
|  | β-actin | BA3R | Mouse IgG2b | Purified | abm |

**Supplementary Table 2. List of primers used for real-time PCR analyses of the indicated genes.**

| Protein | Primer (5'-3') |
| --- | --- |
| MICA | Forward: CCT TGG CCA TGA ACG TCA GG  Reverse: CCT CTG AGG CCT CGC TGC G |
| MICB | Forward: ACC TTG GCT ATG AAC GTC ACA  Reverse: CCC TCT GAG ACC TCG CTG CA |
| ULBP1 | Forward: ATC AGC GCC TCC TGT CCA C  Reverse: AAA GAC AGT GTG TGT CGA CCC AT |
| ULBP2 | Forward: AAA TGT CAC AAC GGC CTG G  Reverse: TGA GGG GTT CCT TGG GTG T |
| ULBP3 | Forward: CGA TTC TTC CGT ACC TGC TAT TCG  Reverse: ATT CTT CTG ATC CAC CTG GCT CT |
| β-actin | Forward: ACT CCA TCA TGA AGT GTG ACG  Reverse: CAT ACT CCT GCT TGC TGA TCC |
