# Supplementary figures and images for: GSK-3α Inhibition in Drug-Resistant CML Cells Promotes Susceptibility to NK Cell-Mediated Lysis in an NKG2D- and NKp30-Dependent Manner
Source: Cancers (Basel). 2021 Apr 9;13(8):1802. doi: 10.3390/cancers13081802 (PMC8070516; doi:10.3390/cancers13081802)

**Fig 2A**

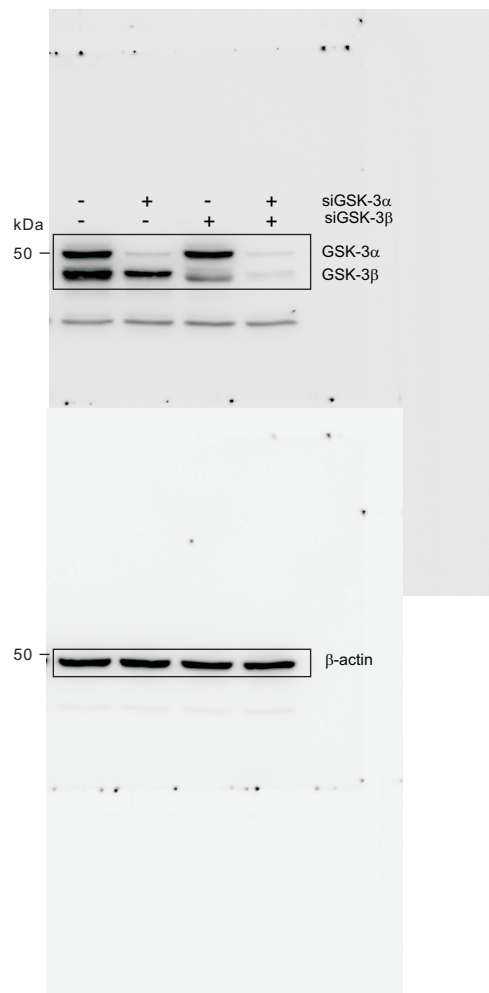

**Fig 3A**

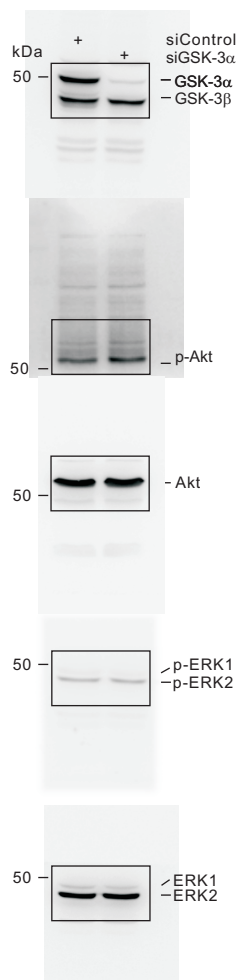

**Fig 3D**

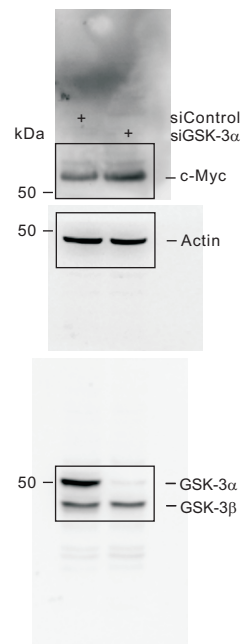

**Fig 4A**

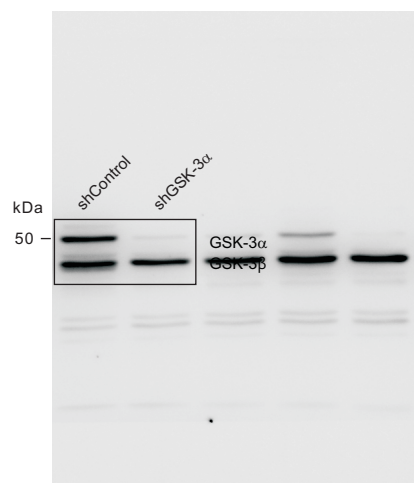

Supplement: Supplementary file 1 [file cancers-13-01802-s001.zip › cancers-1124798_original Images for blots-Proof.pdf]
